# Locking Plate with Cerclage Wiring Versus Hook Plate Fixation for Unstable Distal Clavicle Fractures: Is There Still a Role for Hook Plates?

**DOI:** 10.3390/medicina61101882

**Published:** 2025-10-21

**Authors:** Hyun Seok Song, Hyungsuk Kim

**Affiliations:** Department of Orthopedic Surgery, Eunpyeong St. Mary’s Hospital, College of Medicine, The Catholic University of Korea, Seoul 03312, Republic of Korea; hssongmd@catholic.ac.kr

**Keywords:** clavicle, fractures, bone plate, fracture fixation, postoperative complication

## Abstract

*Background and Objectives*: Hook plate fixation has been widely used for unstable distal clavicle fractures, but concerns remain regarding implant-related complications and the need for secondary removal. Locking plate fixation with supplementary cerclage wiring has been proposed as an alternative that may provide stability while reducing complications. This study compared the clinical and radiologic outcomes of locking plate fixation with cerclage wiring versus hook plate fixation. *Materials and Methods*: A retrospective review was performed on patients who underwent open reduction and internal fixation for unstable distal clavicle fractures (Cho’s classification type II) between 2015 and 2024. Patients with at least 6 months of follow-up were included. Two techniques were evaluated: locking plate with cerclage wiring (Group 1) and hook plate fixation (Group 2). Clinical outcomes, including complications, range of motion, and patient satisfaction, were compared at the final follow-up. *Results*: A total of 52 patients met the inclusion criteria: 27 in Group 1 and 25 in Group 2. The overall mean follow-up period was 13.17 ± 8.46 months. The distribution of fracture types was not significantly different between groups (*p* = 0.287). Complications were more frequent in Group 2 (40%), including postoperative stiffness requiring capsular release (70%), nonunion requiring revision (20%), and peri-implant fracture (10%). The overall union rate was 100% in Group 1 and 80% in Group 2. In contrast, Group 1 had only one complication (3.7%), a peri-implant fracture (*p* = 0.002). Shoulder range of motion at the final follow-up showed no significant difference between groups. *Conclusions*: Hook plate fixation was associated with a significantly higher complication rate compared with locking plate fixation with cerclage wiring. Locking plate fixation with supplementary cerclage wiring appears to be a better surgical option for unstable distal clavicle fractures.

## 1. Introduction

Distal clavicle fractures account for approximately 10–30% of all clavicle fractures and are less common than midshaft fractures [1,2,3]. However, they remain clinically significant because of their higher risk of nonunion. It has been reported that 30–45% of all clavicle nonunion fractures occur distally [4,5]. This risk is particularly pronounced in unstable fracture patterns, such as type II fractures according to Cho’s classification [4]. While stable distal clavicle fractures may be managed conservatively, nonoperative treatment of unstable fractures has been associated with a high rate of nonunion. The nonunion rate has been reported to be as high as 63%, and a substantial proportion of these cases required delayed surgical intervention [6]. Consequently, surgical fixation is generally recommended for unstable distal clavicle fractures to achieve reliable union and restore shoulder function.

Various surgical techniques have been introduced for the management of unstable distal clavicle fractures, including precontoured locking plate fixation, coracoclavicular fixation using suture anchors or button devices, tension band wiring, transacromial pinning, and hook plate fixation [4]. Among these, hook plate fixation has been widely adopted due to its ability to provide stable fixation, particularly in cases with small lateral fragments or concurrent coracoid fractures, as well as its relative ease of use in surgical practice [7]. Although high union rates have been reported with this method, the use of hook plates is frequently associated with complications. These include subacromial impingement, painful postoperative stiffness, acromial erosion, and peri-implant fractures, many of which need implant removal after fracture healing [8,9,10,11]. Furthermore, some studies have reported that a significant proportion of athletes treated with hook plates were unable to return to sports, raising concerns regarding the functional outcome of this technique [12].

Locking plate fixation with supplementary cerclage wiring has also been utilized as a viable surgical option for unstable distal clavicle fractures. Anatomically contoured locking plates allow for multiple screws to be placed in the lateral fragment, and additional cerclage wiring can enhance construct stability in cases with limited bone stock [13]. In particular, in fracture types such as type IID, where an inferior fragment remains without complete rupture of the coracoclavicular ligament, cerclage wiring facilitates effective reduction and stabilization of the fragment. This technique offers the advantage of avoiding subacromial irritation and eliminates the need for routine implant removal. However, despite its clinical relevance, there is a relative paucity of studies directly comparing the clinical outcomes and complication profiles of locking plate fixation with cerclage wiring to those of hook plate fixation. This may be attributed not only to the relatively low incidence of distal clavicle fractures and the predominance of single-technique reports, but also to the fact that clavicle fractures, in general, have historically received less research attention compared with other major fractures. Nevertheless, distal clavicle fractures are by no means simple injuries, as surgical management is technically demanding and requires careful consideration to achieve stable fixation and reliable healing [14,15].

The purpose of this study was to compare the clinical and radiologic outcomes of distal clavicle locking plate fixation with supplementary cerclage wiring and hook plate fixation in the treatment of unstable distal clavicle fractures. We hypothesized that the locking plate with cerclage wiring would yield comparable clinical outcomes to hook plate fixation while resulting in a lower complication rate.

## 2. Materials and Methods

The study protocol was approved by the Institutional Review Board of Eunpyeong St. Mary’s Hospital (No. will be given after acceptance), which waived the requirement for informed consent due to the retrospective design.

### 2.1. Patient Enrollment

This retrospective study included patients with unstable distal clavicle fractures (Cho’s classification type II) who underwent open reduction and internal fixation at a single tertiary university hospital between January 2015 and December 2024. Cho’s classification was used to categorize distal clavicle fractures, as it defines type II fractures as unstable injuries with ≥5 mm displacement and further subdivides them into IIA–IID based on fracture location and coracoclavicular ligament attachment. This system corresponds to Neer type II fractures but provides a more detailed subdivision, allowing for a more precise description of fracture morphology relevant to surgical decision-making [4]. Two surgical techniques were used: distal clavicle locking plate fixation with cerclage wiring (locking plate group) and hook plate fixation (hook plate group). After excluding patients with less than 6 months of follow-up or a prior history of trauma or surgery involving the ipsilateral shoulder, eligible patients were selected for inclusion. All surgeries were performed by board-certified orthopedic surgeons specialized in shoulder surgery at our institution (surgeon initials will be provided after acceptance). The exclusion criteria were open fractures, polytrauma, pathological fractures, age younger than 18 years, and insufficient follow-up duration.

### 2.2. Surgical Procedures

Each patient underwent open reduction and internal fixation under general anesthesia in the supine position. A transverse skin incision was made over the fracture site, followed by meticulous soft tissue dissection. After confirming the fracture pattern, reduction was performed under direct visualization, preserving the integrity of the acromioclavicular (AC) joint.

**Figure 1:**
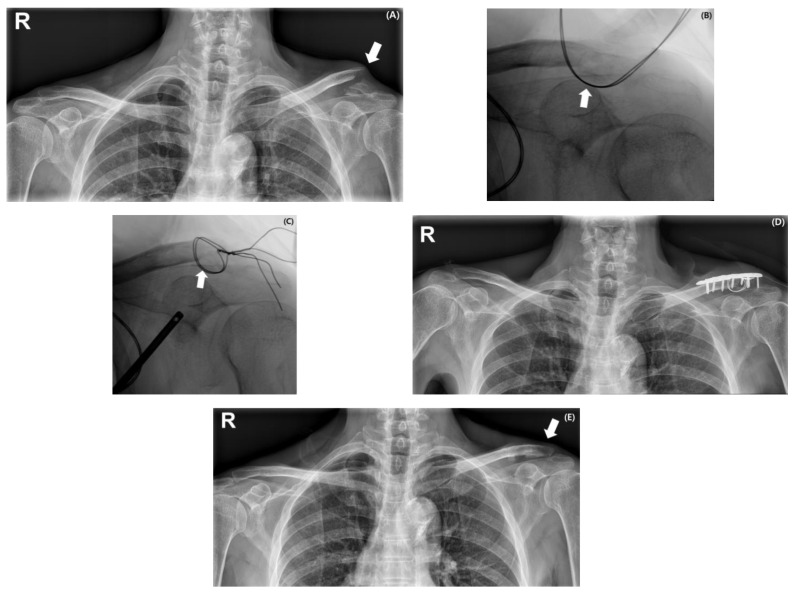
Serial images of a 63-year-old man with a type IID distal clavicle fracture following a fall from height. Preoperative radiograph shows a displaced fracture of the distal clavicle with a prominent medial fracture beak causing skin tenting (indicated by a white arrow) (**A**). Intraoperative fluoroscopic images demonstrate fracture reduction in the inferior fragment achieved using cerclage wiring (indicated by a white arrow) (**B**,**C**). Immediate postoperative radiograph confirms appropriate reduction and stable fixation using a locking plate with cerclage wiring (**D**). At 15 months after implant removal, follow-up radiograph shows maintained reduction and complete healing without complications (indicated by a white arrow) (**E**).

In the locking plate group, cerclage wiring was performed in all included cases. For type IIA and IIB fractures, cerclage wiring was not always mandatory when the lateral fragment was sufficient to accommodate at least three distal locking screws; however, only those patients in whom wiring was performed were included in this group. However, in type IIC and IID fractures, where the lateral fragment was insufficient, the inferior fragment was carefully identified and reduced to the proximal fragment using cerclage wiring. Cerclage wiring was performed using 21-gauge stainless steel monofilament wire in a simple-loop configuration to reduce the inferior fragment to the proximal clavicle. The wire was manually twisted for fixation, and the cut ends were buried to minimize soft-tissue irritation. A distal clavicle locking plate (either from Depuy Synthes Inc., Warsaw, IN, USA or GS Medical Corp., Cheongju-si, Republic of Korea) was then applied over the construct for fixation. In cases with small lateral fragments, the plate was positioned as close as possible to the AC joint to allow insertion of at least two locking screws (Figure 1).

**Figure 2:**
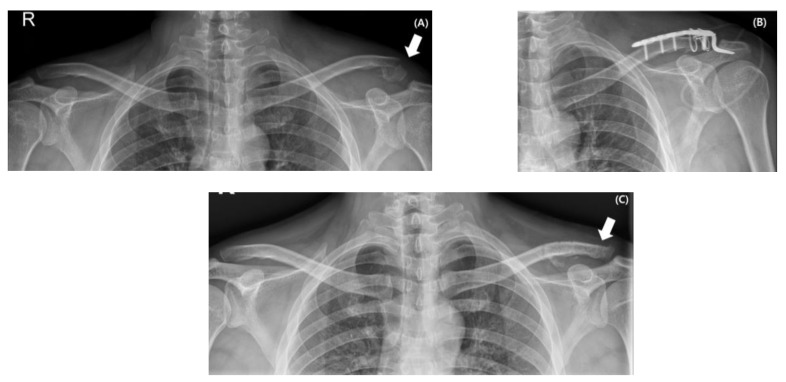
Serial radiographs of a 62-year-old man with a distal clavicle fracture (Type IID) sustained from a fall from height (indicated by a white arrow) (**A**). Surgical treatment involved hook plate fixation with additional cerclage wiring to assist in fracture reduction (**B**). Radiograph obtained at 1 year postoperatively confirmed maintained reduction and complete fracture healing (indicated by a white arrow) (**C**).

In the hook plate group, a hook plate (Depuy Synthes Inc., Warsaw, IN, USA) was inserted beneath the acromion and positioned across the fracture site, extending medially along the clavicle. After provisional fixation with one or two cortical screws, fluoroscopic imaging was used to confirm adequate fracture reduction, appropriate hook depth, and proper plate alignment. Locking screws were then inserted into both the medial and lateral fragments to complete the fixation. Occasionally, cerclage wiring was additionally applied to assist fracture reduction. Coracoclavicular ligament repair was not performed, but the deltotrapezial fascia was meticulously closed over the implant to reinforce the construct (Figure 2).

Postoperatively, all patients were immobilized in a Velpeau sling for four weeks. A gentle range of motion exercise for the hand and elbow was initiated as tolerated during the immobilization period. Return to regular daily activities was allowed beginning at six weeks after surgery.

### 2.3. Clinical and Radiologic Assessment

Clinical assessments were performed at 2, 4, 6, and 8 weeks postoperatively, and at 3 months, 6 months, and 1 year. Shoulder range of motion, including forward flexion, abduction, external rotation at the side, internal and external rotation in abduction, and internal rotation behind the back, was recorded by a physician assistant. Shoulder abduction was measured as pure active glenohumeral abduction, with 90° considered the normal range, consistent with prior reports [16,17,18]. For statistical analysis, internal rotation behind the back was converted to numerical values based on vertebral levels as follows: levels above T12 were assigned a score of 5, T12–L1 as 4, L2–L3 as 3, L4–L5 as 2, and levels below the sacrum as 1 [19,20,21].

Radiologic follow-up included standardized anteroposterior radiographs of both clavicles obtained at the same intervals—2, 4, 6, and 8 weeks postoperatively, and at 3 months, 6 months, and 1 year. In the hook plate group, implant removal was routinely performed in all patients after radiographic union, typically at a mean of 4–5 months postoperatively, because long-term retention of the hook plate is associated with implant-related complications such as subacromial erosion or peri-implant fracture. Patients who did not show radiographic evidence of union by 12 weeks were monitored monthly until removal was deemed appropriate. If postoperative stiffness accompanied by pain was observed prior to removal, conservative management with supervised ROM exercise was first attempted; however, if symptoms persisted, arthroscopic capsular release was performed concurrently with hook plate removal. Final ROM values used for analysis were recorded at the last follow-up after any secondary interventions, including implant removal and/or capsular release. Bone union was defined as either the presence of bridging callus across the fracture site or the absence of a visible fracture line. All radiographs were reviewed to determine time to union and to identify procedure-related complications, including loss of reduction, implant failure, peri-implant fracture, infection, and nonunion. All radiographs were independently assessed by two experienced orthopedic shoulder specialists to determine time to union and procedure-related complications.

### 2.4. Statistical Analysis

All statistical analyses were performed using IBM SPSS Statistics ver. 24.0 (IBM Corp., Armonk, NY, USA). To determine the normal distribution of continuous data, a Kolmogorov–Smirnov test was performed. Student’s *t*-test was used to compare continuous variables between the two groups. Pearson’s chi-square test, Fisher’s exact test, and the linear-by-linear association test were used to compare categorical variables, including demographic characteristics. Mean, standard deviation, and standard error of the mean were calculated for all relevant variables. A *p*-value of <0.05 was considered statistically significant.

## 3. Results

### 3.1. Patient Demographics

A total of 56 patients underwent surgical fixation for unstable distal clavicle fractures during the study period. After excluding four patients with less than 6 months of follow-up, 52 patients were included in the final analysis. Of these, 27 patients were treated with locking plate fixation with cerclage wiring (Group 1), and 25 patients underwent hook plate fixation (Group 2).

There were no significant differences between the two groups in terms of mean age, follow-up duration, sex, affected side, smoking status, or mechanism of injury (Table 1). Smoking was reported in 7 patients (25.9%) in Group 1 and 8 patients (32.0%) in Group 2. Low-energy injuries were observed in 10 patients (37.0%) in Group 1 and 11 patients (44.0%) in Group 2. These injuries were defined as simple falls from standing height. High-energy injuries occurred in 17 patients (63.0%) and 14 patients (56.0%), respectively, and consisted of bicycle accidents, traffic accidents, sports injuries, and falls from height. The overall mean follow-up period was 13.17 ± 8.46 months.

According to Cho’s classification, Group 1 included 8 type IIA fractures (29.6%), 5 type IIB (18.5%), 1 type IIC (3.8%), and 12 type IID (48.1%). Group 2 included 3 type IIA fractures (12.0%), 9 type IIB (36.0%), 2 type IIC (8.0%), and 11 type IID (44.0%). The distribution of fracture types did not differ significantly between the two groups (*p* = 0.694) (Table 1).

### 3.2. Clinical and Radiologic Outcomes

**Figure 3:**
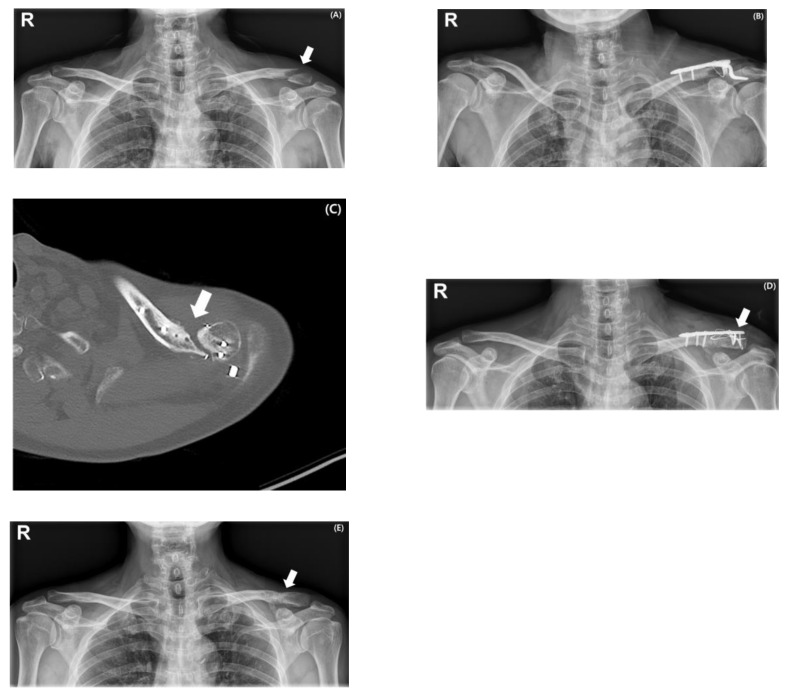
Serial radiographs and an axial CT image of a 59-year-old man with a displaced distal clavicle fracture (Type IIB) following a fall from height (indicated by a white arrow) (**A**). Initial treatment involved hook plate fixation with additional cerclage wiring (**B**). At 3 months postoperatively, an axial CT image demonstrated a persistent fracture gap prior to planned implant removal (indicated by a white arrow) (**C**). Revision surgery was performed using a distal clavicle locking plate with cerclage wiring and tension band wiring (indicated by a white arrow) (**D**). Radiograph obtained at 3 years post-revision confirmed complete fracture union, following implant removal, which had been performed at 1 year post-revision (indicated by a white arrow) (**E**).

The overall complication rate was significantly higher in Group 2 than in Group 1 (40.0% vs. 3.7%, *p* = 0.002). In Group 2, postoperative stiffness was the most common complication, occurring in 7 patients (70%) and requiring arthroscopic capsular release. The procedure was performed in cases where painful limitation of motion was present, and active forward flexion failed to exceed 120 degrees by 3 to 4 months postoperatively, during the period when implant removal was being considered. To further assess the potential impact of these secondary interventions, a sensitivity analysis was performed, excluding the seven patients who underwent arthroscopic capsular release. This subanalysis showed no significant differences in final shoulder range of motion or functional outcomes between the two groups, confirming that our overall findings were not influenced by secondary interventions. Nonunion was observed in 2 patients (20%), diagnosed when definitive radiographic evidence of union was not present by 6 months postoperatively. Both cases required revision surgery. One patient (10%) sustained a peri-implant fracture following minor trauma (Figure 3). The mean time to radiographic union was 3.65 ± 1.20 months in Group 1 and 4.06 ± 1.89 months in Group 2. The difference between the two groups was not statistically significant (*p* = 0.256).

In contrast, only one complication occurred in Group 1 (3.7%), which was a peri-implant fracture. No cases of postoperative stiffness or nonunion were observed in this group.

Radiographic union was achieved in all patients in Group 1. In Group 2, union was eventually achieved in 23 of 25 patients (92.0%), while the remaining two progressed to nonunion and required revision surgery.

At the final follow-up, there were no statistically significant differences in shoulder range of motion between the two groups. Forward flexion, internal rotation at back, external rotation at the side, abduction, external and internal rotation in abduction, were comparable (Table 2).

## 4. Discussion

The main finding of this study was that the complication rate associated with hook plate fixation was significantly higher than that observed in locking plate fixation with cerclage wiring for unstable distal clavicle fractures. Although both techniques achieved satisfactory radiographic union rates, the hook plate group demonstrated a markedly greater incidence of complications, particularly postoperative stiffness and nonunion requiring additional surgical intervention. In contrast, locking plate fixation with cerclage wiring was associated with a low complication rate while providing stable fixation and facilitating fracture healing. These results support the hypothesis that locking plate fixation with cerclage wiring may serve as a safer and more reliable surgical option for managing unstable distal clavicle fractures.

Various fixation methods have been applied to unstable distal clavicle fractures, including hook plates, locking plates (with or without coracoclavicular augmentation), CC stabilization using suture-buttons, T-plates, and tension band wiring. Although no single technique is universally accepted, recent systematic reviews suggest that locking plate fixation, especially when combined with cerclage wiring or CC stabilization, and suture-button techniques may offer superior safety and comparable efficacy to hook plates. A meta-analysis by Panagopoulos et al. involving 790 patients across 34 studies concluded that locking plate fixation with or without CC augmentation and coracoclavicular stabilization techniques demonstrated better clinical outcomes and fewer complications than hook plate fixation [22]. Similarly, Uittenbogaard et al. reported that after analyzing 2284 patients that hook plate fixation led to lower Constant–Murley scores and higher revision rates than other fixation techniques, while locking plate and CC techniques had more favorable profiles [14]. Kim et al. reviewed clinical outcomes of various fixation techniques for distal clavicle fractures and highlighted that pre-contoured locking plates generally achieved high union rates with few complications, particularly when the lateral fragment allowed for stable fixation. In cases of minimal lateral fragment, adjunct techniques such as cerclage wiring or button fixation have been used to reinforce stability effectively [4]. Furthermore, prospective comparative studies such as the one by Teimouri et al. comparing hook plates to T-plates demonstrated similar functional outcomes and union rates at six months, but noted better early ROM and pain scores in the T-plate group, with lower hardware irritation and absence of major complications [23]. These findings are further supported by recent high-level comparative data. In the prospective RCT, although different from our study in that distal locking plate fixation was combined with CC fixation, Orlandi et al. reported that union rates at 12 months were 100% in both the hook plate and locking plate with CC fixation groups. However, patients treated with a locking plate with CC augmentation demonstrated quicker early recovery as reflected in DASH improvements from 6 to 12 months and a trend toward superior Constant-Murley shoulder scores [24]. In addition, the meta-analysis by Li et al. found that locking plates achieved significantly better Constant-Murley scores at 3 and 6 months, with fewer cases of shoulder pain and limited abduction compared to hook plates, though rates of delayed union and subacromial impingement did not differ significantly [25].

Hook plate fixation has long been favored for unstable distal clavicle fractures due to its technical simplicity, its immediate rigid fixation properties, and its utility in cases with small or comminuted distal fragments that may not accommodate multiple screws. Because the hook engages beneath the acromion, it obviates the need for direct fixation into the often-fragile lateral segment and offers stable leverage with consistent outcomes, particularly in cases with limited lateral bone stock or comminution [26]. However, despite these mechanical advantages, hook plate fixation is consistently associated with a significant complication burden. Several clinical studies have highlighted the complication risks associated with hook plate fixation. In a retrospective study involving 160 patients, Lee et al. reported a complication rate of 43.1%, with painful shoulder stiffness being the most common issue. Subacromial erosion was also frequently observed and described as an unavoidable outcome of the hook plate design [9]. Similarly, Beisemann et al. found a complication rate of 20.8% in their cohort, including deep infection, nonunion, and peri-implant fractures [27]. Singh et al. noted that hook plate removal was necessary in 62.5% of cases due to implant-related irritation, a rate substantially higher than with locking plate constructs [28].

In the current study, the complication rate in Group 2 (hook plate) was 40.0%, consistent with previous reports. Among these, postoperative stiffness requiring arthroscopic capsular release occurred in 7 patients (70%), nonunion in 2 patients (20%), and a peri-implant fracture in 1 patient (10%). In contrast, Group 1 (locking plate with cerclage wiring) had only one complication (3.8%), a peri-implant fracture; no cases of stiffness or nonunion were observed. These findings reinforce concerns regarding the complication burden associated with hook plate fixation and support the use of alternative fixation strategies that avoid placing hardware beneath the acromion and reduce the need for routine implant removal.

This study has some limitations. First, its retrospective design may have introduced selection bias and limited the control over confounding variables. Second, although the sample size was relatively small, a post hoc power analysis was conducted using a chi-square test based on the observed difference in complication rates between the two groups (3.8% vs. 40%), with a significance level (α) of 0.05. The analysis yielded a statistical power of 1.00, indicating that the sample size was adequate to detect a significant difference. Third, functional outcomes were evaluated based on range of motion measurements rather than a standardized scoring system. While this limits direct comparison with prior studies, we believe that detailed ROM assessment served as a clinically relevant alternative for evaluating postoperative function in this comparative study. Finally, we acknowledge that Cho’s classification, although providing greater anatomical detail, is not yet widely known or universally adopted compared with the Neer or AO systems. This may limit the generalizability of our findings.

The strengths of this study are as follows. First, we used Cho’s classification system, which provides greater anatomical specificity and clinical relevance than the conventional Neer classification. Recent literature has emphasized the need for refined or novel subclassifications in complex upper limb injuries, such as Monteggia fracture–dislocations, where rare variants have been described that fall outside the established Bado or Jupiter systems [29]. This growing trend underscores the importance of continuous reassessment of existing classification systems to ensure they reflect clinical realities and can better guide treatment strategies. In this context, our findings contribute to validating the clinical relevance of Cho’s classification for distal clavicle fractures. Second, we directly compared a relatively novel technique—locking plate fixation with cerclage wiring—with conventional hook plate fixation in the treatment of unstable distal clavicle fractures. Third, all surgeries were performed at a single tertiary institution by fellowship-trained shoulder specialists, minimizing inter-surgeon variability. Fourth, functional outcomes were objectively evaluated using a detailed range of motion measurements, providing a quantifiable assessment of postoperative recovery.

## 5. Conclusions

Hook plate fixation was associated with a significantly higher complication rate compared with locking plate fixation with cerclage wiring. Locking plate fixation with supplementary cerclage wiring appears to be a better surgical option for unstable distal clavicle fractures.

## Figures and Tables

**Table 1:** Patient demographic data.

Variable	Group 1 (n = 27)	Group 2 (n = 25)	*p*-Value
Mean age (years)	50.96 ± 15.69 (range: 28–76)	49.56 ± 17.25 (range: 20–69)	0.760
Follow-up duration (months)	14.41 ± 5.63 (range: 12–36)	11.84 ± 10.82 (range: 12–42)	0.283
Sex			0.427
Male	21 (77.8%)	17 (68%)	
Female	6 (22.2%)	8 (32%)	
Affected side			0.376
Right	13 (48.1%)	9 (36%)	
Left	14 (51.9%)	16 (64%)	
Smoking status	7 (25.9%)	8 (32%)	0.629
Mechanism of injury			0.609
Low energy	10 (37%)	11 (44%)	
High energy	17 (63%)	14 (56%)	
Cho’s classification			0.694
IIA	8 (29.6%)	3	
IIB	5 (18.5%)	9	
IIC	1 (3.8%)	2	
IID	13 (48.1%)	11	

**Table 2:** Clinical assessment results.

Variable	Group 1 (n = 27)	Group 2 (n = 25)	*p*-Value
Complication	1 (3.7%)	10 (40%)	**0.002**
Non-union	0 (0%)	2 (20%)	
Postoperative stiffness	0 (0%)	7 (70%)	
Peri-implant fracture	1 (100%)	1 (10%)	
Time to union (months)	3.65 ± 1.20	4.06 ± 1.89	0.256
Range of motion at final follow-up			
Forward flexion	169.62 ± 2.42	169.20 ± 1.87	0.497
Internal rotation at back	4.04 ± 0.45	4.64 ± 0.35	0.570
Side external rotation	76.92 ± 7.49	72.80 ± 8.79	0.077
Abduction	94.04 ± 2.46	92.20 ± 4.81	0.096
Abduction external rotation	81.73 ± 8.36	77.20 ± 9.90	0.083
Abduction internal rotation	70.38 ± 3.72	70.80 ± 5.72	0.759

## Data Availability

The raw data supporting the conclusions of this article will be made available by the authors on request.
